# Alloying hBN with aluminum influences absorption and electronic properties

**DOI:** 10.1038/s41598-025-92671-9

**Published:** 2025-03-22

**Authors:** Jakub Iwański, Mateusz Tokarczyk, Aleksandra K. Dąbrowska, Jan Pawłowski, Piotr Tatarczak, Marcin Strawski, Kamil Sobczak, Marta Bilska, Maciej Wójcik, Sławomir Kret, Johannes Binder, Andrzej Wysmołek

**Affiliations:** 1 Faculty of Physics, University of Warsaw, Pasteura 5, 02-093 Warsaw, Poland; 2 Laboratory of Electrochemistry, Faculty of Chemistry, University of Warsaw, Pasteura 1, 02-093 Warsaw, Poland; 3 Biological and Chemical Research Centre, Faculty of Chemistry, University of Warsaw, Żwirki i Wigury 101, 02-089 Warsaw, Poland; 4 Institute of Physics, Polish Academy of Sciences, Aleja Lotników 32/46, 02-668 Warsaw, Poland

**Keywords:** BAlN alloy, hBN, Epitaxy, MOVPE, Bandgap manipulation, Excitonic absorption, Surfaces, interfaces and thin films, Two-dimensional materials, Electronic properties and materials

## Abstract

The versatile range of applications for two-dimensional (2D) materials has encouraged scientists to engineer their properties. This is often accomplished by stacking atomically thin layered materials into complex van der Waals heterostructures. A less popular but technologically promising approach is alloying 2D materials. In this work, we demonstrate a first step towards tuning the intrinsic electronic properties of hexagonal boron nitride (hBN). We present a series of aluminum alloyed hexagonal boron nitride (hBAlN) samples grown by metal organic vapor phase epitaxy on 2-inch sapphire substrates with varying aluminum concentration. Importantly, the obtained samples revealed a sp^2^-bonded crystal structure and modifications in interband optical transitions. Optical absorption experiments disclosed two prominent peaks in the excitonic spectral range with absorption coefficients ~ 10^6^ cm^− 1^. Their peak energies align closely with the energies of indirect and direct bandgap transitions in hBN. The presence of two absorption peaks can be attributed to mixing of electronic states in the K and M conduction band valleys, resulting in a substantial increase in the absorption coefficient for indirect transitions. The observed effects offer insights into hBN-based two-dimensional alloys, highlighting the potential for developing 2D material-based quantum well structures capable of operating in the challenging deep UV spectral range.

**Supplementary Information:**

The online version contains supplementary material available at 10.1038/s41598-025-92671-9.

## Introduction

The initial research on graphene has motivated researchers to explore the properties of other atomically thin layered materials (2D materials) and their stacks, sometimes referred to as NanoLego structures^1–4^. This is typically accomplished by stacking different layers together into van der Waals heterostructures^5,6^. Combining these materials using epitaxial growth provides the additional opportunity to scale-up or create new structures by growing materials as alloys with appropriate composition^7,8^. In the case of hexagonal boron nitride (hBN), the natural choice is alloying hBN with aluminum and gallium, since they are in the same group of the periodic table as boron. Research on the novel hB_*1 − x−y*_Al_*x*_Ga_*y*_N material (where *x* and *y* stand for Al and Ga concentration in the alloy respectively) is interesting in terms of exploration of new physical phenomena as well as from a technological point of view^9^. hBN with its, unusual for III-N, strong sp^2^ covalent bonds in plane and weak van der Waals bonds out of plane exhibits extraordinary properties^10^. One of the intriguing facts is that in spite of the indirect character of the bandgap, hBN shows a photoluminescence (PL) emission intensity of the phonon-assisted transitions as strong as for direct semiconductors^11–13^. Thus, the possibility of tuning the hBN bandgap using Al opens up further prospects for applications in hBN-based quantum wells and optoelectronic devices. The same scheme could be used for hBN alloying with Ga. Tunable hBAlN, hBGaN or hBAlGaN alloys could become promising candidates for building blocks of efficient light sources in the deep UV spectral range (DUV, 200–280 nm, 4.4–6.2 eV). Such candidates are of high importance, since nowadays the efficiency of semiconductor light sources operating in DUV is very limited (~ 10% for 270–280 nm and ~ 3% for 250 nm)^14^. This spectral range is crucial, for example for the disinfection and sterilization of air, water and surfaces^15,16^.

However, before hBN-based quantum structures and DUV light sources can be realized, more fundamental questions have to be addressed. Firstly, it is still unclear how to tune the hBN bandgap energy. This knowledge is of great importance for designing hBN-based quantum wells that could trap carriers and enhance emission from the structure. Moreover, it would be very beneficial for further improvement if one could change the nature of the hBN bandgap from indirect to direct^17–19^.

In this work, we present results for boron nitride layers grown by metal organic vapor phase epitaxy (MOVPE) that were alloyed with aluminum and preserve a sp^2^-bonded crystal structure. The produced hB_*1 − x*_Al_*x*_N layers with aluminum concentration ranging from *x* = 0.01% to *x* = 1.1% exhibit strong absorption for two energies that coincide with energies of indirect and direct bandgap in hBN. Our approach that uses MOVPE to grow this novel material provides a perspective for further development of the hBAlN alloy composition control in terms of doping for conductivity, manipulation of the bandgap, and commercial up-scaling.

## Methods

### Samples

The samples used in these studies were grown using an Aixtron CCS 3 × 2’’ metal organic vapor phase epitaxy system. The growth was carried out on 2-inch sapphire *c*-plane substrates with 0.3° off-cut. For the growth of layered, sp^2^-bonded hB_*1 − x*_Al_*x*_N samples ammonia, triethylboron (TEB) and trimethylaluminum (TMAl) were used as nitrogen, boron, and aluminum precursors and hydrogen was used as carrier gas. Note that we used the hBAlN notation (with letter h) to highlight the importance of the hexagonal hBN-like crystal structure of the obtained material. The precursors were injected alternatively following the flow modulation epitaxy growth mode^20^. The scheme of pulses in a single cycle was as follows: TEB-NH_3_-TMAl-NH_3_. The growth temperature was kept at 1300 °C. The temperature value was controlled with an in situ multipoint optical pyrometer (ARGUS). NH_3_ and TEB flows were fixed for all samples (V/III ratio for TEB-NH_3_ pulses was ~ 15 000). The TMAl/III ratio (III = TEB + TMAl) in the process varied for the samples: Al_0.02_ (ratio 0.02), Al_0.04_ (ratio 0.04), Al_0.07_ (ratio 0.07), Al_0.13_ (ratio 0.13). To obtain the lowest ratio, the duration of consecutive pulses of TMAl and NH_3_ was reduced by half. This step was enforced by the minimal precursor flow limitation in our system. The number of pulsing cycles was chosen in such a way that all samples were grown 60 min.

### Experimental details

The crystal structure of the samples was examined with a Panalytical X’Pert diffractometer with standard CuKα X-ray source. The X-ray beam was formed by an X-ray mirror for Bragg-Brentano optics. Infrared reflectance spectra were collected with a Thermo Scientific Nicolet Continuum Infrared Microscope equipped with a 32x Schwarzschild infinity corrected objective (NA 0.65). All samples of 7 × 10 mm were measured with a perpendicular incident beam on five 70 × 70 μm areas placed in the center and four corners of the sample. Spectra were collected in the range of 650–4000 cm^− 1^ with a resolution of 0.482 cm^− 1^. The surface morphology was examined by scanning electron microscopy (SEM) using a FEI Helios NanoLab 600 system. This device equipped with gallium focus ion beam was also utilized to prepare electron-transparent cross sections of the samples for transmission electron microscopy (TEM). The sample surfaces were protected with electron-deposited Pt-C composite from a metalorganic source and underwent plasma cleaning before measurements. High-resolution TEM images were acquired using the FEI Titan 80–300 transmission electron microscope operating at 300 kV. Composition mapping was obtained using the FEI Talos F200X microscope operating at 200 kV equipped with energy-dispersive X-ray spectroscopy detector (Bruker). Scanning transmission electron microscopy (STEM) compositional maps were captured within a scattering angle range of 80 to 200 mrad and a converged semi-angle of 9.5 mrad for the incident beam. The secondary ion mass spectrometry (SIMS) depth profiling experiment was conducted using the TOF-SIMS.5 system (ION-TOF GmbH, Germany), equipped with a time-of-flight mass analyzer (TOF-SIMS). Samples were analyzed without special pretreatment upon receipt, and they were transferred directly to the analytical chamber, where the pressure was maintained at 9 × 10^− 10^ mbar. Elemental distribution was acquired using the dual beam mode, with the samples being sputtered by oxygen ions (operating conditions: 250 eV, 23 nA) over a 250 μm × 250 μm area. Subsequently, the internal layers of the sample were analyzed using a Bi^3+^ ion beam (operating conditions: 64 μm × 64 μm raster size, 30 keV, 0.5 pA). Internal mass calibration was performed using the mass of ions that are consistently present: B_*x*_O^+^ and Al_*x*_O^+^ clusters. Absorption spectra were measured using a Varian Cary 5000 UV-vis-NIR spectrophotometer in dual-beam mode with nitrogen purging. The spectral bandwidth was set to 1 nm.

Although our samples are very homogeneous on the wafer scale^21,22^, we decided to measure each of the samples taken from the exact same area on the wafer.

## Results

### Material composition

**Fig. 1:**
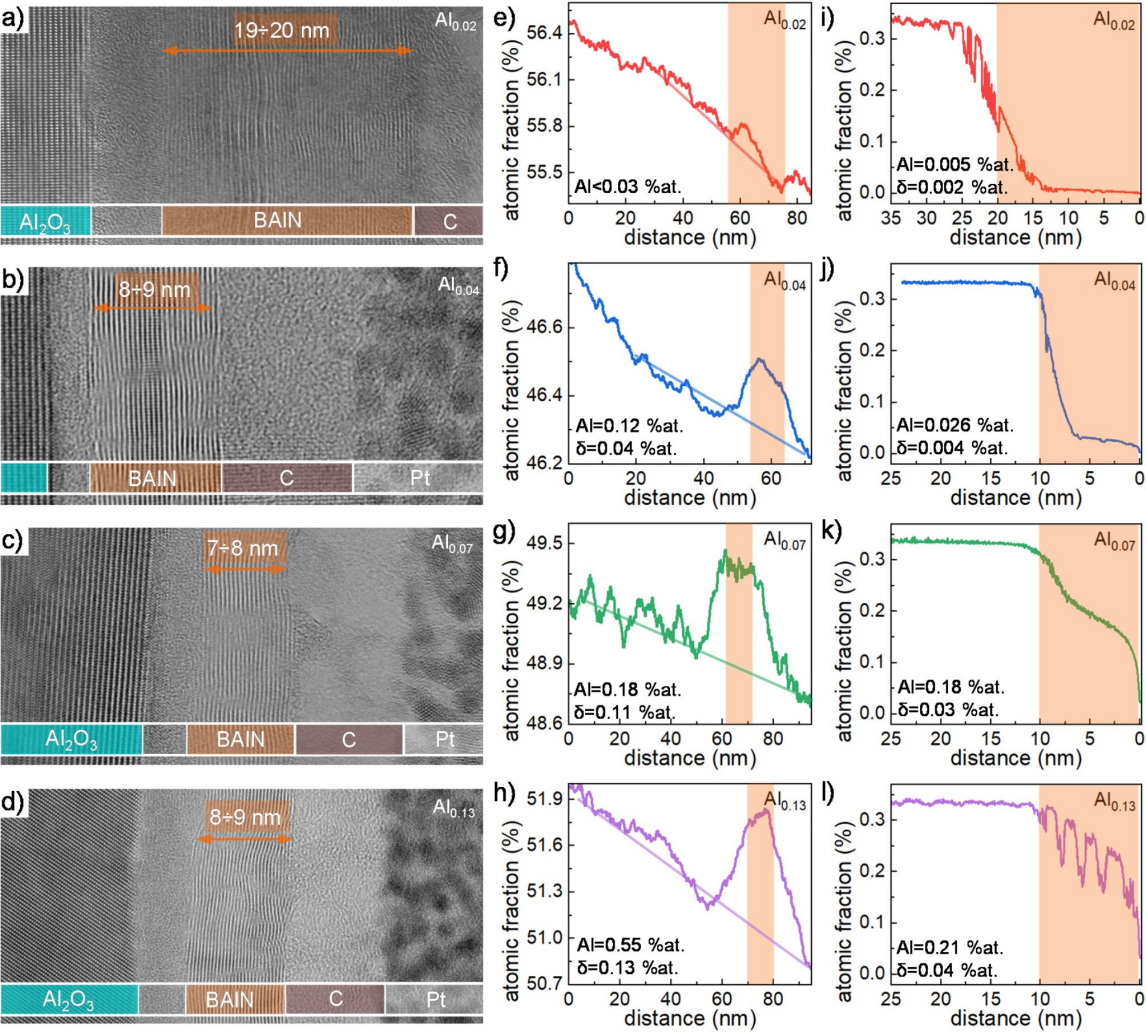
Cross-sectional high-resolution transmission electron microscopy pictures for samples (**a**) Al_0.02_, (**b**) Al_0.04_, (**c**) Al_0.07_, (**d**) Al_0.13_. Carbon (C) and platinum (Pt) caps were deposited in lamella preparation process. Atomic fraction profiles derived as integrated signal from energy-dispersive X-ray spectroscopy (EDX) maps for samples (**e**) Al_0.02_, (**f**) Al_0.04_, (**g**) Al_0.07_ (**h**) Al_0.13_. The distance on horizontal axis refers to map coordinate across the sample. Straight lines visualize trend for Al_2_O_3_ background signal. Secondary ion mass spectrometry (SIMS) results for aluminum ion for samples (**i**) Al_0.02_, (**j**) Al_0.04_, (**k**) Al_0.07_, l) Al_0.13_. The distance on horizontal axis refers to the distance from sample interface. Orange rectangle in (**e**)-(l) visualizes signal corresponding to BAlN layer. Al values denote to aluminum composition extracted from the measurement and δ is its uncertainty.

High-resolution transmission electron microscopy (HRTEM) images for each sample are presented in Fig. 1a-d. The BAlN layers are observed to consist of atomic planes aligned parallel to the sapphire substrate characteristic for epitaxial growth of layered, two-dimensional materials^22^. At the Al_2_O_3_-BAlN interface, there is an unintentional, transitional layer of approximately 2 nm thickness composed of amorphous AlON. This layer forms during the initial stages of growth, prior to the hBAlN layer covering and protecting the substrate from ammonia exposure as described in the literature^21,23^. Additionally, above the BAlN layer, there are carbon and platinum cap layers deposited during the process of sample preparation for TEM measurements. Material composition was assessed using energy-dispersive X-ray spectroscopy (EDX) mapping in cross-sectional scanning transmission electron microscopy (STEM) measurements (see Supplementary Materials). As depicted in Fig. 1e–h, an excess of aluminum signal is observed in the EDX map area corresponding to BAlN, superimposed on a monotonous background originating from the Al_2_O_3_ substrate signal. Upon background signal subtraction, aluminum composition values were obtained, correlating with the amount of TMAl in the gas phase during the growth process. Based on the EDX results, the estimated compositions for the grown samples are as follows: below 0.03% for Al_0.02_, 0.12% for Al_0.04_, 0.18% for Al_0.07_, and 0.55% for Al_0.13_. Assuming aluminum substitution for boron in the crystal lattice, we infer a nitrogen concentration of 50% at. Consequently, based on EDX results, the composition of hB_*1 − x*_Al_*x*_N ranges from *x* < 0.06% to *x* = 1.1%. Comparing the ratio of metalorganic precursors to the resulting material composition, we deduce an aluminum incorporation efficiency of only 3–8%.

Alternatively, we conducted secondary ion mass spectrometry (SIMS) measurements to independently determine the aluminum composition of the as-grown samples. To do this, we assumed that the value derived from the EDX result for sample Al_0.07_ applies similarly to the SIMS measurement. This assumption enabled us to calculate the efficiency of Al^+^ ion spreading during sample milling. We selected sample Al_0.07_ because its EDX signal is robust and clearly distinguishable from the background, while the SIMS profile is smoother compared to sample Al_0.13_, which shows a stronger EDX signal. Subsequently, the obtained signal for Al^+^ ions for the remaining samples was recalculated to atomic fraction. The results are presented in Fig. 1i–l. The plateau observed at 0.35% atomic composition arises from the saturation of the detector, resulting in its nonlinear response. Consequently, we are unable to accurately monitor aluminum composition above 0.35% atomic. It is evident that for samples Al_0.02_ and Al_0.13_, the profiles exhibit raggedness. This is attributed to sample charging during measurement. The aluminum composition values obtained for the samples differ from those obtained through EDX analysis because we operate in very small concentrations regime. Nonetheless, the trends remain consistent. According to the SIMS results, we estimate aluminum composition as follows: 0.005% for Al_0.02_, 0.026% for Al_0.04_, 0.18% for Al_0.07_, and 0.21% for Al_0.13_. Applying the same assumption as in the case of EDX results to the SIMS data, we find that the material composition ranges from *x* = 0.01% to *x* = 0.42%, with an estimated efficiency of aluminum incorporation of approximately 0.5-5%.

Both experimental methods exhibit varying detection sensitivity within this range of atomic composition. Additionally, the obtained results are interrelated, as the composition derived for one sample was utilized to calibrate the results for others. Consequently, pinpointing the exact composition for our samples is challenging. Nevertheless, it is evident that we are operating within a compositional range with aluminum concentrations not exceeding 1%.

### X-ray diffraction

**Fig. 2:**
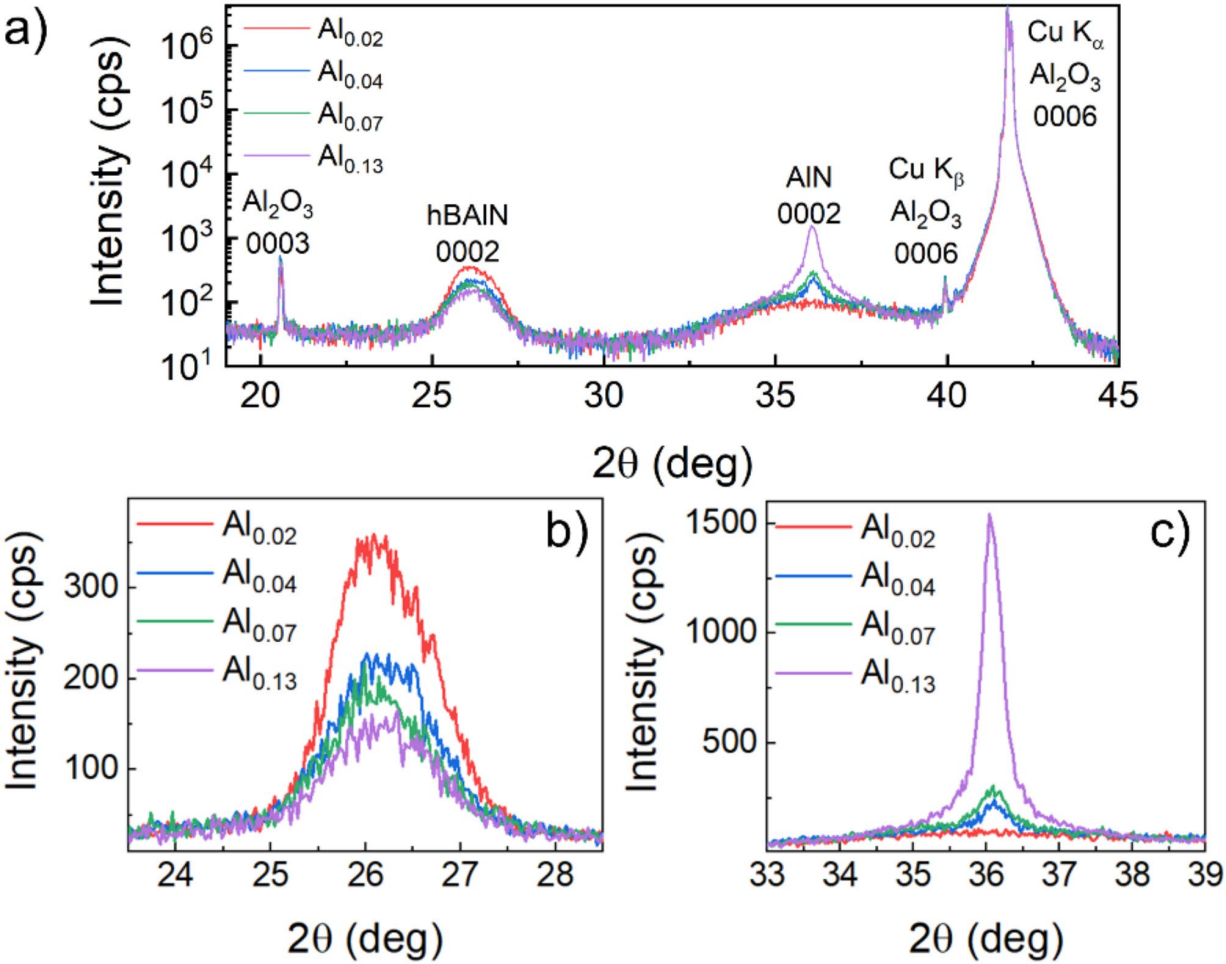
X-ray diffraction 2θ\ω scans of the studied samples: (a) scan in a wide 2θ range (semi-log scale), (b) zoom on the peak related to hBAlN and (c) zoom on the peak related to AlN.

In Fig. 2a we present X-ray diffraction 2θ/ω scans collected for all the studied samples. The peaks at 20.5°, 40° (Cu K_β_ line) and 41.75° (Cu K_α_ line) correspond to the 0003 and 0006 planes of Al_2_O_3_. In Fig. 2b, c we present a zoom on the peaks at 26° and 36° that come from the reflection of the 0002 planes in sp^2^-BAlN and sp^3^-AlN, respectively. The parameters of the Gaussian curve fit performed on the data concerning hBAlN are included in Table 1. Note that the 0002 BAlN peak related to sample Al_0.02_ is asymmetric, so we fitted two Gaussian curves yielding two different components of the peak. The components originate from turbostratic (tBAlN, lower angles) and hexagonal (hBAlN, higher angles) phases of sp^2^-bonded BAlN^21,23^. In the further analysis we will focus only on the hexagonal phase. In Table 1 we do not observe significant changes in the peak position 2θ_B_ and the full width at half maximum (FWHM) for the hBAlN peak. This implies that the *c* lattice constant is comparable for all the samples (~ 3.41 Å) as well as the thickness of the crystal. The only monotonous trend can be found in the amplitude of the hBAlN peak. This means that the misorientation of the 0002 crystal planes is enlarged when increasing the TMAl flow.

**Table 1:** Parameters of the Gaussian curve fit to 0002 BAlN XRD peak. For sample Al_0.02_ two Gaussian curves were fitted, indicating the presence of both tBAlN and hBAlN.

Sample	Amplitude (cps)	2θ_B_ (deg)	FWHM (deg)
Al_0.02_	7.0(6)	25.84(1)	0.51(4)
	28.2(5)	26.18(8)	1.28(1)
Al_0.04_	18.5(2)	26.13(7)	1.29(1)
Al_0.07_	15.0(2)	26.07(9)	1.31(2)
Al_0.13_	11.4(2)	26.12(3)	1.36(2)

The signal associated to AlN consists of a very broad peak, which is alike for all the samples and a narrower component that changes its intensity. In fact, these two components have different origins. The broad component comes from an unintentional thin (~ 2 nm) AlON layer that is created on the hBAlN-sapphire interface during the initial growth stage. This layer is also observed in HRTEM results in Fig. 1a-d. This kind of layer is characteristic for MOVPE-grown hBN^23,24^. However, in accordance with the enhancement of the sharp component with an increase in TMAl, we can deduce the formation of AlN crystals in our samples. These crystals do not seem to influence the crystal structure of hBAlN as we do not observe any correlation between the intensity of the sharp peak at 36° and the values of 2θ_B_ and FWHM in Table 1 which are related to BAlN.

### Fourier-transform infrared spectroscopy

**Fig. 3:**
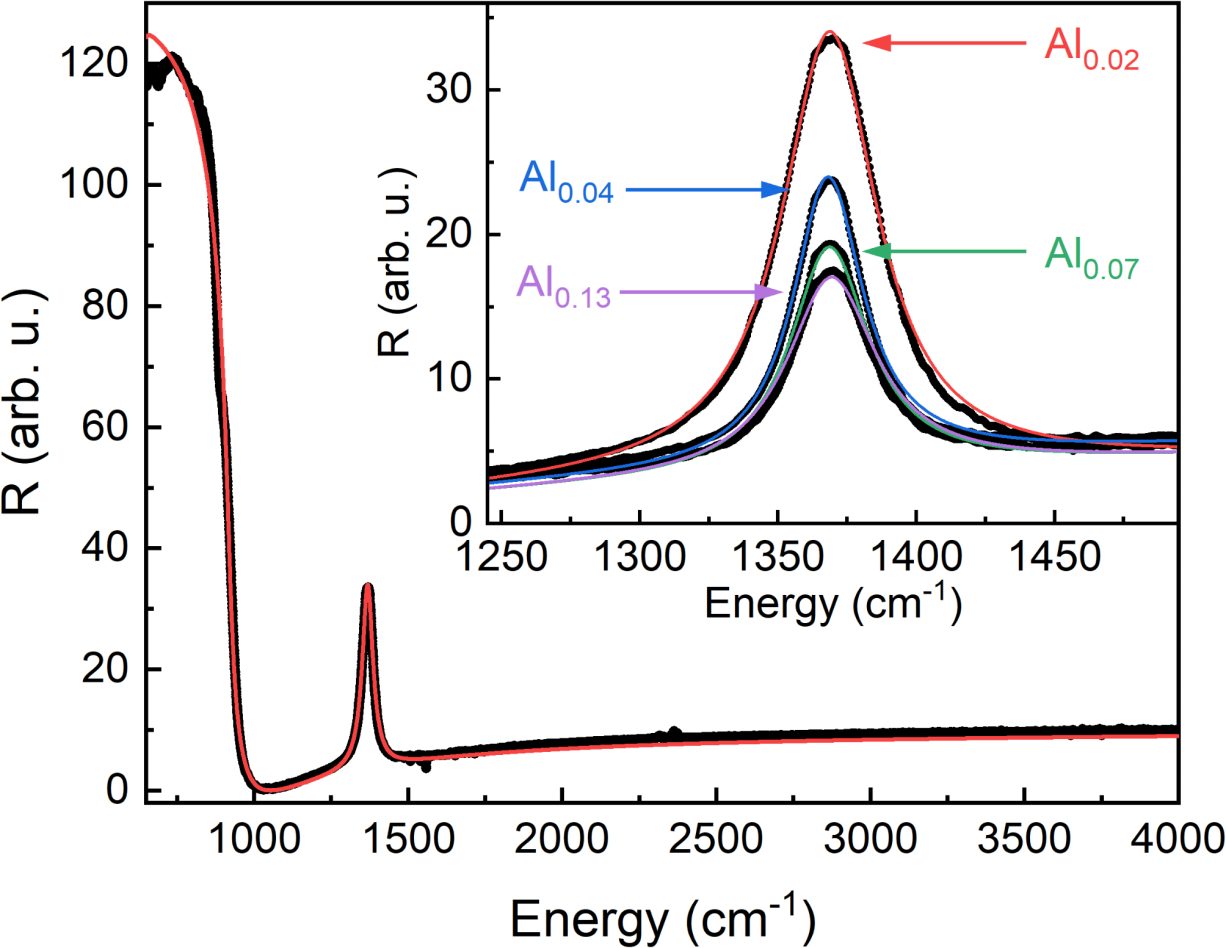
Fourier-transform infrared reflectance spectrum for the Al_0.02_ sample with the lowest TMAl flow (black dots). The red line is the fitted curve. The inset shows a zoom on the peak of the E_1u_ vibrational mode, which is strong evidence for a sp^2^ crystal structure for all hBAlN samples. The black dots represent the experimental data and the lines are the fitted curves: red for Al_0.02_, blue for Al_0.04_, green for Al_0.07_, purple for Al_0.13_.

In Fig. 3 we present exemplary infrared reflectance spectra obtained for the studied samples. The peak observed at approximately 1368 cm^− 1^ corresponds to the E_1u_ vibrational mode characteristic of hBN^25^. The observation of this peak provides direct evidence of the sp^2^ bonding in our samples. FTIR results for the E_1u_ mode complement the analysis of the E_2g_ Raman mode and offer similar insights into variations of stress within the hBAlN layers. However, the advantage of the FTIR technique lies in the absence of background photoluminescence signals, which can be disruptive in Raman spectroscopy. The high reflectance below 1000 cm^− 1^ comes from the sapphire substrate. Barely visible small and sharp peaks about 1550 cm^− 1^, 2300 cm^− 1^ and 3500 cm^− 1^ correspond to transitions in the atmospheric gases present during the experiment.

To make the FTIR results quantitative, we implemented a spectra analysis method, as described in the Ref^26^. In this method, it is assumed that the material is composed of harmonic oscillators with self-energy ω and damping parameter γ. It makes use of the Dynamic Dielectric Function (DDF) of the materials in the structure (boron nitride and sapphire in this case). The substantial advantage of this method is the possibility of characterizing the grown layer itself. The self-energy of the oscillator ω_BAlN_ (phonon energy, peak position), its damping parameter γ_BAlN_ (peak broadening) and layer thickness provide information about strain, homogeneity and growth rate. In Table 2 we present the best fit parameters obtained as an average of 5 points measured for each sample.

**Table 2:** Averages of the best fit parameters of the FTIR spectra obtained for the studied samples.

Sample	ω_BAlN_ (cm^− 1^)	γ_BAlN_ (cm^− 1^)	d_BAlN_ (nm)
Al_0.02_	1368.9(5)	24.5(1)	18.6(5)
Al_0.04_	1368.0(3)	22.1(2)	9.1(4)
Al_0.07_	1368.7(2)	26.5(6)	9.8(3)
Al_0.13_	1369.3(2)	30.1(5)	10.1(3)

The fitted parameters are monotonous for the samples Al_0.04_, Al_0.07_ and Al_0.13_. The increase in TMAl flow leads to an increase in phonon energy and peak broadening, which suggest the introduction of an inhomogeneous compressive strain within the layer. The changes of ω_BAlN_ (~ 0.63 cm^− 1^ for the following samples) and γ_BAlN_ (~ 4 cm^− 1^ for the following samples) are significant in terms of FTIR measurements. Remarkably different is the trend for the layer thickness. The 4-time increase in amount of TMAl (samples Al_0.04_ and Al_0.13_) resulted in a layer that is only 1 nm thicker, which is equivalent to the increase of the growth rate by less than 0.02 nm/min.

The sample Al_0.02_ clearly stands out and does not follow the same trends. The reason for this behavior is most likely due to the change of TMAl-NH_3_ pulse duration necessary to obtain a TMAl/III ratio equal to 0.02. This led to a two-fold increase in growth rate compared to the sample Al_0.04_ as can be seen in doubled value of the layer thickness. This result is reasonable, since the TMAl/III ratio for Al_0.02_ was twice lower than for Al_0.04_ yielding to increase of material synthesis rate. At the same time the number of TEB pulses increased by 33% aiming for uniform growth duration across all samples. So we find the competition between TEB-NH_3_ and TMAl-NH_3_ pulses to be more important for the growth rate rather than the change in number of TEB pulses in the growth process. This finding also opens up avenues for studying the effectiveness of aluminum incorporation depending on the timing of precursor injection.

### Morphology analysis

**Fig. 4:**
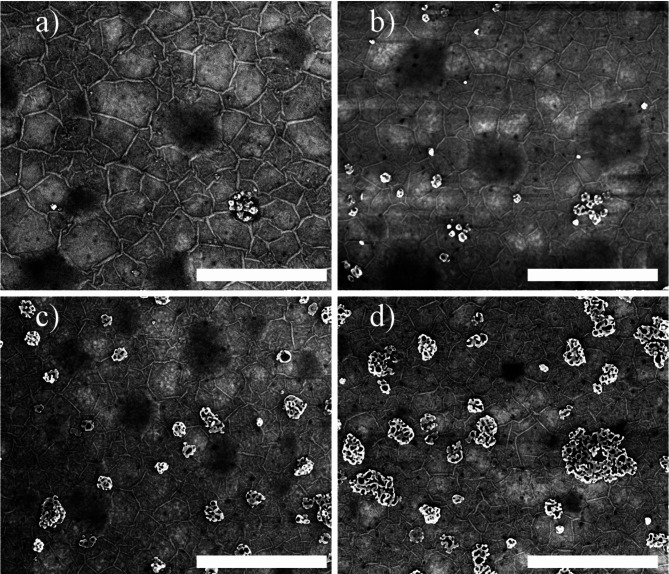
Scanning electron microscopy images of the samples (**a**) Al_0.02_, (**b**) Al_0.04_, (**c**) Al_0.07_, (**d**) Al_0.13_. The scale bar corresponds to 1 μm for each image.

The morphology of the studied samples is presented in Fig. 4. For each sample, a characteristic wrinkle pattern is observed. The wrinkles are created during cooling down the material after the growth process and are due to the relaxation of strain caused by the difference in thermal expansion coefficients of the layer and the substrate^26,27^. Because of the mechanism of the wrinkle formation their presence is an evidence for the continuity of the hBAlN epitaxial layer. The wrinkles are most pronounced for sample Al_0.02_, which is due to a much larger thickness. The darker circular areas in the images are bubbles with hydrogen inside. They are created during SEM imaging and are the result of the radiolysis of interfacial water via electron irradiation^28^. The size of crystalline objects on the surface of the layer is correlated with the amount of TMAl present in the process. This observation is in good agreement with the XRD results for which the AlN narrow diffraction peak was observed for Al_0.04_, Al_0.07_ and Al_0.13_.

Additionally, supported by atomic force microscopy (AFM) and nanomechanical mapping, we can rule out the possibility that the crystallites are other forms of boron nitride. Furthermore, Raman spectroscopy revealed a signal originating from the E_2_^high^ characteristic mode in wurtzite aluminum nitride, with its intensity correlating with the number of crystalline objects observed in SEM. Additionally, wurtzite precipitations were observed in HRTEM images. All of these findings, detailed in the Supplementary Materials, indicate that the crystalline objects observed in SEM are sp^3^-bonded aluminum nitride. This poses challenges in aluminum incorporation into hBN, as BN and AlN, being stable in nature, exhibit significantly different allotropic phases. However, in EDX analysis, we selected areas without sp^3^-bonded AlN inclusions, suggesting that aluminum may also incorporate into the sp^2^-bonded crystal structure.

### UV-vis spectroscopy

**Fig. 5:**
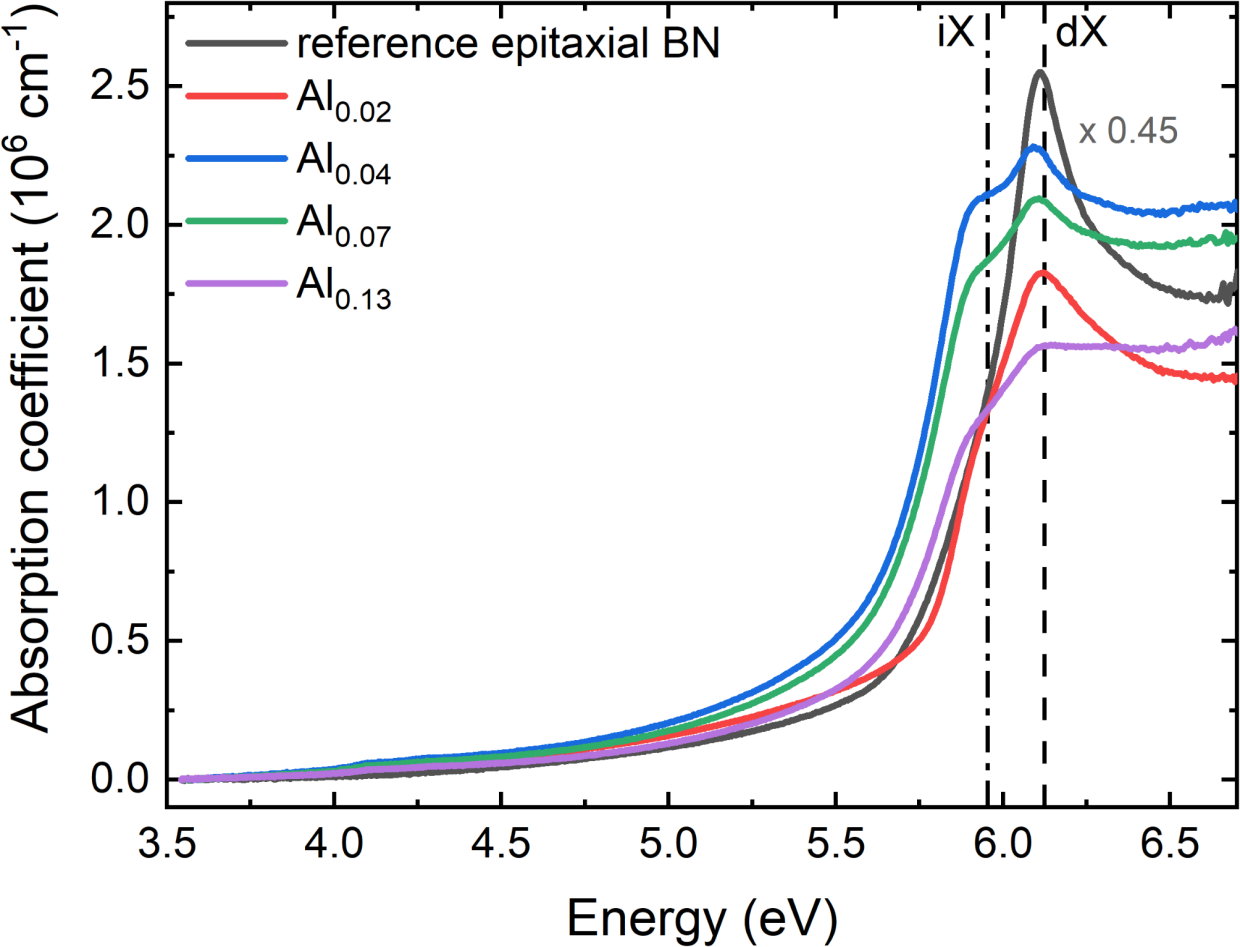
Absorption coefficient spectra of the samples studied. The solid gray line is the spectrum collected for the reference boron nitride sample without aluminum. It was multiplied by factor 0.45 for clarity. The reference sample was grown in analogy to the S^I^ from Ref^21^. The uncertainty of the results is of order of 0.5 × 10^5^ cm^− 1^. Black dashed and dash-dotted lines illustrate the energies of direct and indirect bandgaps in boron nitride.

To determine the absorption coefficient from the measured absorbance, we subtracted the absorbance spectrum obtained for bare sapphire. The thickness of the layer, *d*_BAlN_, extracted from Table 2, was used to calculate the absorption coefficient. The fitted values of *d*_BAlN_ align well with the layer thickness observed in HRTEM (Fig. 1). Additionally, in FTIR measurements, we averaged the thickness over a much wider area in comparison to HRTEM results. Therefore, we consider these values as the best approximation of the average thickness necessary for interpreting the absorption spectra. The absorption coefficient spectra of the samples studied are presented in Fig. 5. The spectrum for the reference epitaxial boron nitride without aluminum sample was obtained in the same way. The reference sample is a thin BN layer grown at 1300 °C in continuous flow growth (CFG) regime analogously to the sample S^I^ presented in the work in Ref^21^. The absorption coefficient for all the samples is of order of α = 2 × 10^6^ cm^− 1^. This value is twice higher as compared to those previously reported for boron nitride^10,29,30^ which most likely originates from high crystalline quality of the material. In contrast to the reference sample, the samples with aluminum exhibit two well-resolved peaks. In previous studies only peak shifting was observed, which was accompanied by a broadening due to a decrease of sample quality^31^. In the case of our samples the two peak energies are close to the dash-dotted and dashed lines that are positioned at energies corresponding to the indirect (5.955 eV^32^) and direct (6.125 eV^33,34^) band-edge transitions in boron nitride, respectively. Depending on the TMAl flow, one can change the intensity of those peaks and the intensity ratio between higher and lower energetic peaks. However, this relationship does not correlate with the amount of TMAl in the growth process or aluminum composition in the BAlN layer.

The lower energy peak, which is not observed for the reference sample, could be thought to be the result of the crystalline objects presented in the SEM images (Fig. 4). However, the peak intensity does not scale with the number of objects on the layer’s surface. Secondly, as has been mentioned before, the crystallites are identified as sp^3^-bonded AlN which is known to have a bandgap energy of 6.2 eV^35^. Possible alloying of sp^3^-bonded wurtzite-AlN (wAlN) with boron would, on the other hand, further increase the bandgap since wurtzite-BN (wBN) is a wider bandgap semiconductor^18^. As a result, the signal from AlN should be observed at higher, rather than lower energies when compared to the peak of pure boron nitride. To ensure that the observed effect is not induced by crystalline objects, we removed them by etching with NaOH. The NaOH solution dissolves AlN without damaging the BAlN layer. No changes were detected in the spectrum within the wAlN bandgap energy region (6.2 eV), which can be attributed to the significantly lower absorption coefficient of this material. After the removal of the crystallites, the lower-energy peak remains present, corroborating that the observed effect originates from the layer itself (see Figure S9).

## Discussion

As presented in Fig. 2b; Table 1, the X-ray diffraction peak is observed at angles lower than expected for the 0002 hBN plane (26.764°^36^). This indicates a larger lattice constant in the *c* direction. However, the peak position does not change significantly between the samples, which is in contrast to the results reported in Ref^31^. in which authors observed peak position shifting towards higher angles with an increasing TMAl flow. According to theoretical DFT calculations presented in Ref^37^. , a decrease in the *c* lattice constant should be observed when hBN is alloyed with Al, since hexagonal aluminum nitride has calculated a smaller *c* lattice constant. We conclude that since we do not have a perfect hexagonal phase the main reason for the XRD peak position change in our samples is that it is attributed to random twists of subsequent atomic layers, which make the material more turbostratic. In this case we postulate that the peak position variations related to the alloying with small amounts of Al are just a higher order correction. This hypothesis seems to be confirmed by the fact that all samples were grown under the same growth conditions. Another prominent feature presented in Fig. 2c is the peak at ~ 36°, whose intensity increases with the increase in TMAl flow. The value of the peak position is in agreement with the XRD peak of 0002 AlN in the crystal structure of wurtzite^38^. This indicates the creation of sp^3^-bonded wAlN crystals, which scale with the amount of TMAl. This additional notable peak is a proof of the phase separation of sp^2^-bonded hBAlN and sp^3^-bonded wAlN. This conclusion is further supported by SEM results (Fig. 4), observation of E_2_^high^ peak related to wAlN in Raman spectroscopy (Fig. S6) and wurtzite structure precipitations observed in HRTEM (Fig. S8). The number and size of crystalline objects correlate with the amount of TMAl, which provides strong evidence for the very limited solubility of aluminum in the hexagonal boron nitride layer at the temperature and pressure used in the growth process (1300 °C, 400 mbar). Nevertheless, using cross-sectional STEM with EDX and SIMS we were able to determine aluminum concentration within hB_*1 − x*_Al_*x*_N layers (*x* < 1%). By comparison of TMAl in a gas phase during growth process and final material composition we estimate aluminum incorporation rate to be approximately 3%.

Although AlN crystallites can be observed, the sp^2^-bonded, layered structure of hBAlN is maintained, which is proved by HRTEM and FTIR measurement presented in Figs. 1a–d and 3. However, by introducing TMAl we modify the optical properties of the layer. This can be seen as a shift of the phonon energy ω_BAlN_ towards higher energies that suggests compressive stress in the structure as demonstrated in Ref^39^. Furthermore, the increase in TMAl is followed by a broadening of the peak described by the parameter γ_BAlN_, which indicates a defect-related inhomogeneity of the strain within the layer.

The most notable finding of this study is the identification of an additional low-energy peak in the absorption spectra. While previous studies have reported features related to indirect bandgap transitions, primarily evidenced by phonon replicas in reflectivity and photoluminescence measurements, our work provides results of a peak in absorption that corresponds well with the expected energy of the indirect excitonic transition. The hBN conduction band is known to consist of minima at the K and M points of the Brillouin zone that are energetically close to each other^11^. They are responsible for direct and indirect band transitions, respectively. As an indirect transition is a three-particle (photon, electron, phonon) event, it has a much lower probability to occur than a direct transition, which requires only two particles (photon, electron). Consequently, the absorption coefficient related to indirect transitions is usually 2–3 orders of magnitude lower as compared to direct ones. However, in the case of our samples, we observe two peaks at energies which coincide with the energies of direct and indirect transitions in hBN (dash-dotted and dashed lines in Fig. 5). Furthermore, both have a very high value of the absorption coefficient (α ~ 10^6^ cm^− 1^) typical for direct bandgap transitions. Notably that the lower-energy peak has even lower energy than expected for the indirect transition in hBN. This observation is in agreement with predictions about a decrease in bandgap energy when hBN is alloyed with Al^17^. To understand the mechanism that stands behind the observation of the two peaks in the absorption spectra, we need to be aware of the role of aluminum in the crystal structure. Al incorporation introduces short range disorder, which in turn would lead to the mixing of states with large *k*-vectors. As presented in Ref^11^. , conduction and valence bands along KH and ML points in hBN are very flat and almost parallel to each other. This feature leads to high oscillator strength and consequently very efficient absorption. A disorder induced by Al incorporation further modifies the band structures and the probability of electronic transitions, i.e. oscillator strength. Consequently, other absorption channels are enabled through the defect-related states. The modification of states caused by Al-related defects allows us to observe highly effective absorption for both energies. However, further increase of TMAl flow and limited solubility of Al in hBN lead to a deterioration of optical quality of the material which is observed in broadening of spectroscopic peaks in the spectra (same for FTIR and UV-Vis). In this case, two opposing effects influence the results: (i) an increase in oscillator strength, which enhances the efficiency of the indirect transition, and (ii) a degradation in optical quality, causing broader and less distinct absorption peaks. As a consequence, the relationship between absorption spectra and aluminum concentration is not straightforward. The optimal balance between these effects was achieved for the sample Al_0.04_.

## Conclusions

In this work, we have shown the results for MOVPE grown hB_*1 − x*_Al_*x*_N layers with TMAl/III ratio ranging from 0.02 to 0.13. High-resolution transmission electron microscopy, X-ray diffraction and Fourier-transform infrared spectroscopy measurements confirmed that the obtained material maintained a sp^2^-bonded layered crystal structure, characteristic for hBN. Composition analysis through energy-dispersive X-ray spectroscopy (EDX) and secondary ion mass spectrometry (SIMS) revealed that our hB_*1 − x*_Al_*x*_N samples have aluminum compositions ranging from *x* = 0.01% to *x* = 1.1%, indicating an effectiveness of aluminum incorporation of approximately 3%. The variations in aluminum composition significantly impact the properties of the layers. Most importantly, we have shown two peaks of strong absorption (α ~ 10^6^ cm^− 1^) typical for direct excitonic transitions in hBN. The peak energies coincide with the energy of the indirect and direct bandgap transition in hBN, indicating the activation of absorption channels through defects introduced by Al incorporation. These findings shed light on the manipulation of the boron nitride bandgap, affecting its indirect/direct nature and width. Such understanding is crucial for fabricating hBN-based structures capable of efficient deep UV emission.

## Electronic supplementary material

Below is the link to the electronic supplementary material.

Supplementary Material 1

## Data Availability

Data for this article, including the experimental data presented in the manuscript are available at the Repository for Open Data RepOD at https://doi.org/10.18150/J8UYQK.
