# Supplementary material for: Alloying hBN with aluminum influences absorption and electronic properties
Source: Sci Rep. 2025 Mar 22;15:9904. doi: 10.1038/s41598-025-92671-9 (PMC11929823; doi:10.1038/s41598-025-92671-9)
Supplement: Supplementary file 1 — Supplementary Material 1 [file 41598_2025_92671_MOESM1_ESM.pdf]

Supplementary Materials: Alloying hBN with aluminum influences absorption and electronic properties

Jakub Iwański,[*,1] Mateusz Tokarczyk,[1] Aleksandra K. Dąbrowska,[1] Jan Pawłowski,[1] Piotr Tatarczak,[1] Marcin Strawski,[2] Kamil Sobczak,[3] Marta Bilska,[1] Maciej Wójcik,[4] Sławomir Kret,[4] Johannes Binder,[1] and Andrzej Wysmołek[1]

[1]*Faculty of Physics, University of Warsaw, ul. Pasteura 5, 02-093 Warsaw, Poland*

[2]*Laboratory of Electrochemistry, Faculty of Chemistry, University of Warsaw, 00-927 Warsaw, Poland*

[3]*Biological and Chemical Research Centre, Faculty of Chemistry, University of Warsaw, PL-02-089 Warsaw, Poland*

[4]*Institute of Physics Polish Academy of Sciences, Aleja Lotnikow 32/46, 02-668 Warsaw, Poland*

* E-mail: Jakub.Iwanski@fuw.edu.pl

STEM-EDX mapping

Aluminum concentration in the analyzed samples was determined using secondary ion mass spectrometry (SIMS) in conjunction with scanning transmission electron microscopy (STEM) mapping supported by energy-dispersive X-ray spectroscopy (EDX). This section provides additional details on the STEM-EDX analysis, which yielded the aluminum concentration values presented in the main text.

For each sample, an electron-transparent cross-sectional lamella was prepared using an FEI Helios NanoLab 600 system equipped with a gallium-focused ion beam. The lamellae were subsequently analyzed using an FEI Talos F200X microscope operating at 200 kV, fitted with a Bruker energy-dispersive X-ray spectroscopy (EDX) detector. The number of compositional STEM-EDX maps acquired varied by sample: 9 maps for $Al_{0.02}$, 11 for $Al_{0.04}$, 6 for $Al_{0.07}$, and 2 for $Al_{0.13}$. The higher number of maps for $Al_{0.02}$ was necessary due to its low aluminum concentration, while additional maps for sample $Al_{0.04}$ were motivated by the presence of the strongest two peaks observed for this sample in optical absorption experiments. Representative STEM-EDX maps for all samples included in this study are shown in Figure S1.

The presented maps reveal a localized reduction in oxygen concentration, indicating the position of the BAlN layer. Within this region, a corresponding increase in aluminum concentration is observed. This effect is particularly pronounced in the $Al_{0.13}$ sample, which has the highest aluminum content. These findings provide clear evidence of aluminum incorporation into the BAlN layer. To quantify this observation, the signal was integrated across the entire map and normalized such that the aluminum concentration in the Al_2O_3 substrate was set to 40%at. The aluminum concentration in the BAlN layer, determined as the excess signal above the substrate background, is shown in figure 1 of the main text. The reported aluminum concentration values and their uncertainties (δ) represent the mean and standard deviation obtained from all measured maps for each sample.

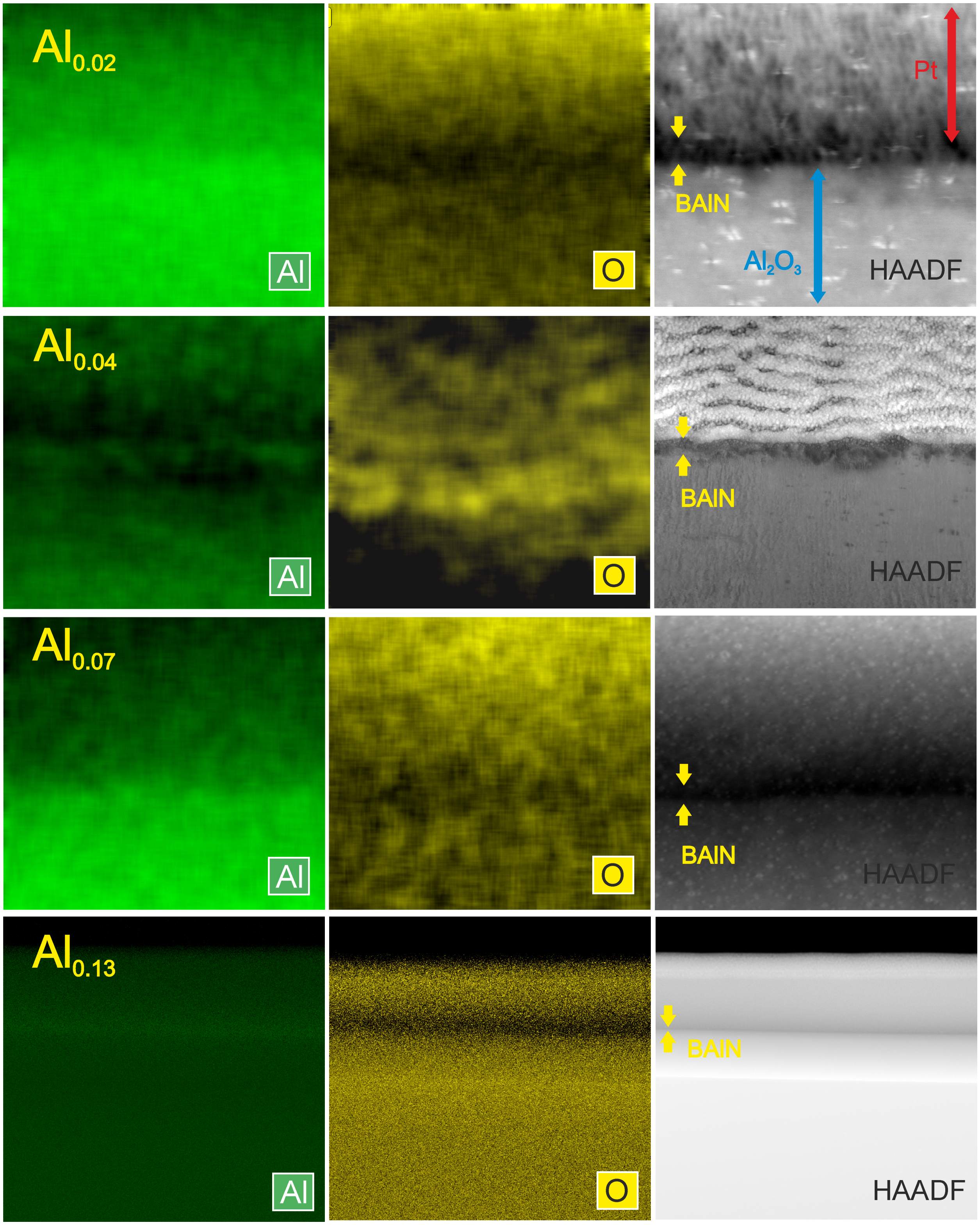

Figure S1: STEM-EDX compositional maps for the samples $Al_{0.02}$, $Al_{0.04}$, $Al_{0.07}$, $Al_{0.13}$. The green intensity map (left column) indicates the spatial distribution of aluminum, while the yellow intensity map (middle column) corresponds to oxygen concentration. Right column presents corresponding HAADF maps with BAlN, Al2O3 and Pt cap positions marked.

AFM characterization of the crystaline objects

Atomic force microscopy (AFM) experiments were performed using Dimension Icon microscope integrated with a NanoScope 6 controller (Bruker Corporation, Billerica, MA, USA). The topography images were collected in Peak Force Tapping™ mode using Bruker RTESPA-300 probes made of antimony n-doped silicone (nominal spring constant of 40 N/m and a resonance frequency of 300 kHz). Nanomechanical mapping was performed using Peak Force Quantitative NanoMechanics™ (QNM) mode. Before each experiment the exact value of spring constant was determined by Sader method. Moreover, deflection sensitivity of the cantilevers and curvature radius of the probes were evaluated using dedicated calibration samples and procedures. All measurements were recorded in air at constant temperature of 22 ± 1 °C.

The samples topography presented in figure S2 shows a result consistent with that of scanning electron microscopy in the main text. The wrinkles characteristic for epitaxial layers are present on all the samples. Number and size of the objects on the hBAlN layer increase with the amount of TMAl in the growth process. The shapes of those objects seem to be more and more complex for samples with higher TMAl flow. Both wrinkles and objects are reproduced figures S3, S4, S5 that present adhesion, deformation and stiffness modulus of the material, respectively. This contrast (between flat hBAlN layer and the objects) that is observed for all AFM images proves presence of two different types of the material. To make data related to hBAlN and crystaline objects easier to compare we calculated average values of adhesion, deformation, Young's modulus. They are collected in table S1. The exact values of the quantities vary for each sample. However, some trends can be easily seen. The hBAlN layers adhese stronger than crystalites which can be due to different nature of bonds of surface atoms. Young's modulus values suggest that hBAlN layers have higher stiffness in comparison to objects on its surface. However, both of them deform comparably under AFM cantilever.

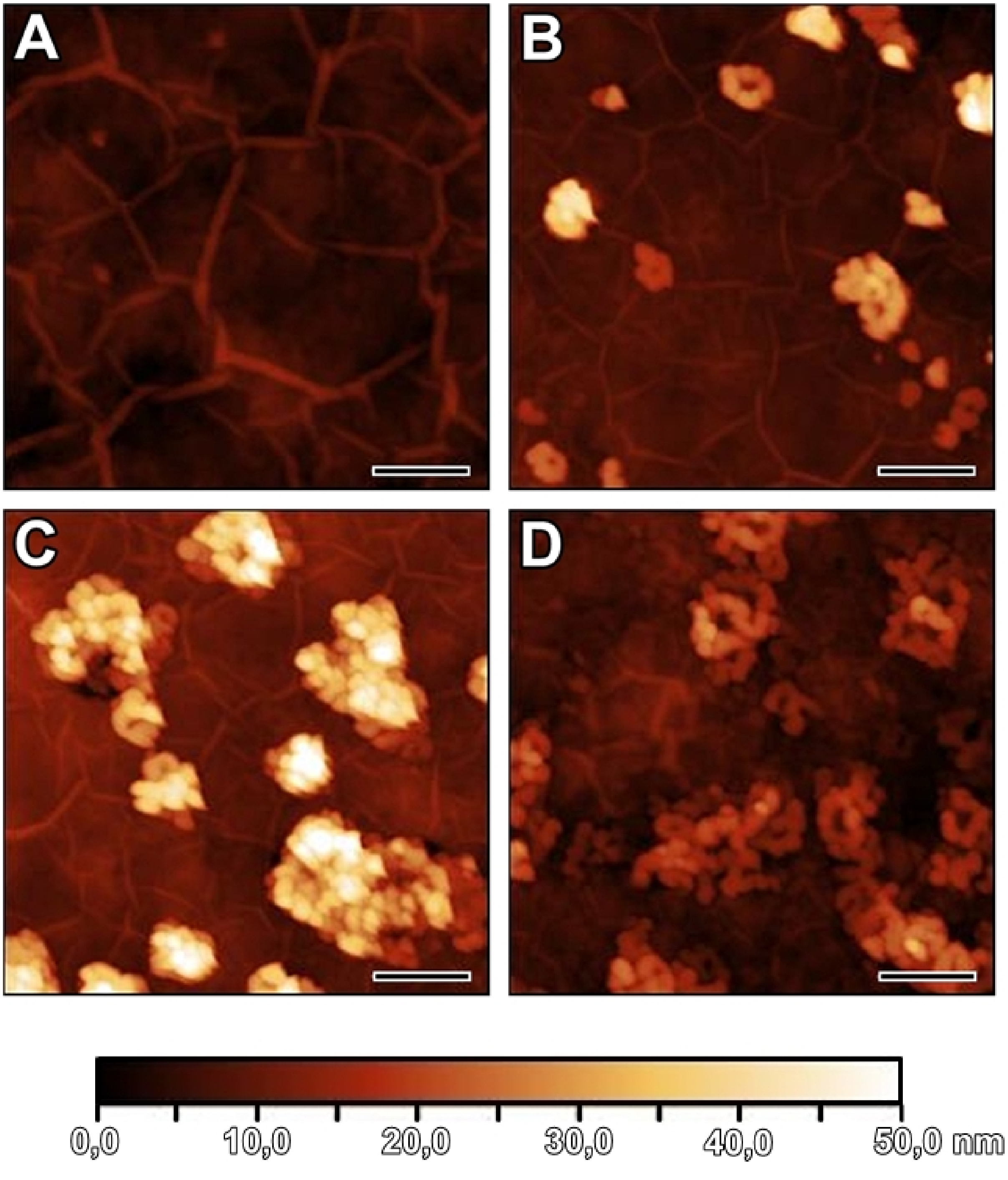

Figure S2: AFM topography images of hBAlN layers grown on sapphire substrate. a) $Al_{0.02}$; b) $Al_{0.04}$; c) $Al_{0.07}$; d) $Al_{0.13}$. The scale bars correspond to 200 nm.

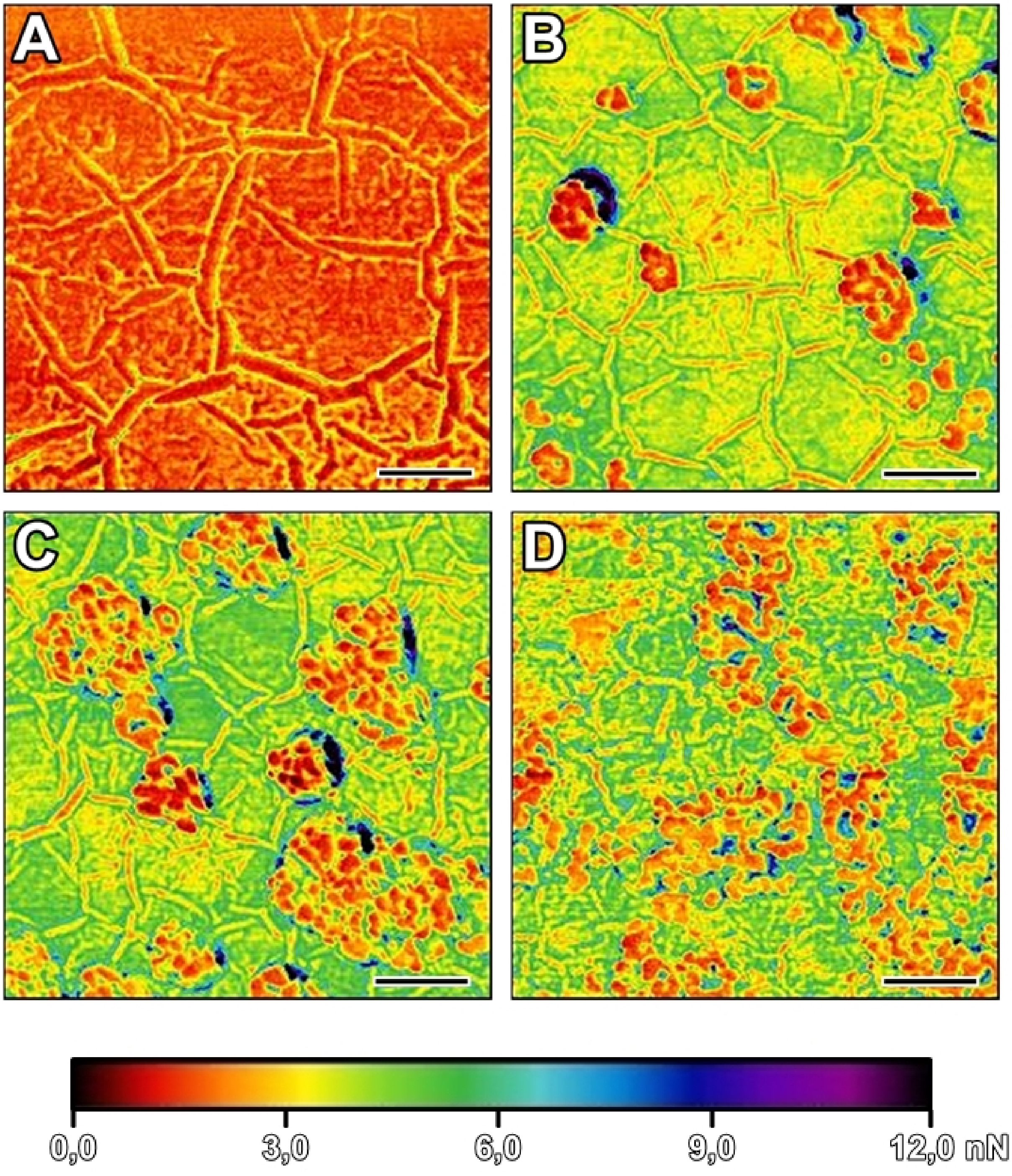

Figure S3: AFM adhesion images of hBAlN layers grown on sapphire substrate. a) $Al_{0.02}$; b) $Al_{0.04}$; c) $Al_{0.07}$; d) $Al_{0.13}$. The scale bars correspond to 200 nm.

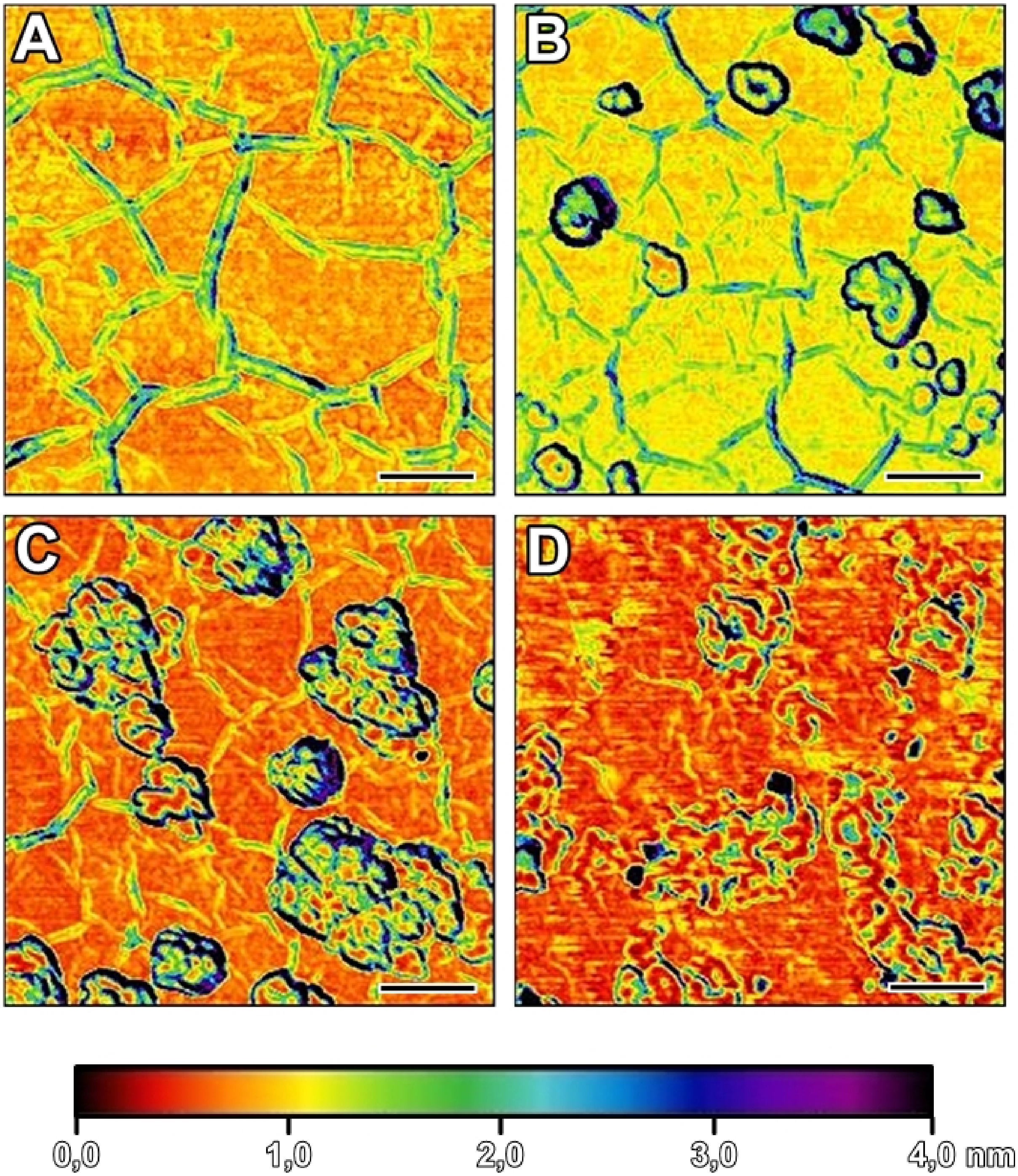

Figure S4: AFM deformation images of hBAlN layers grown on sapphire substrate. a) $Al_{0.02}$; b) $Al_{0.04}$; c) $Al_{0.07}$; d) $Al_{0.13}$. The scale bars correspond to 200 nm.

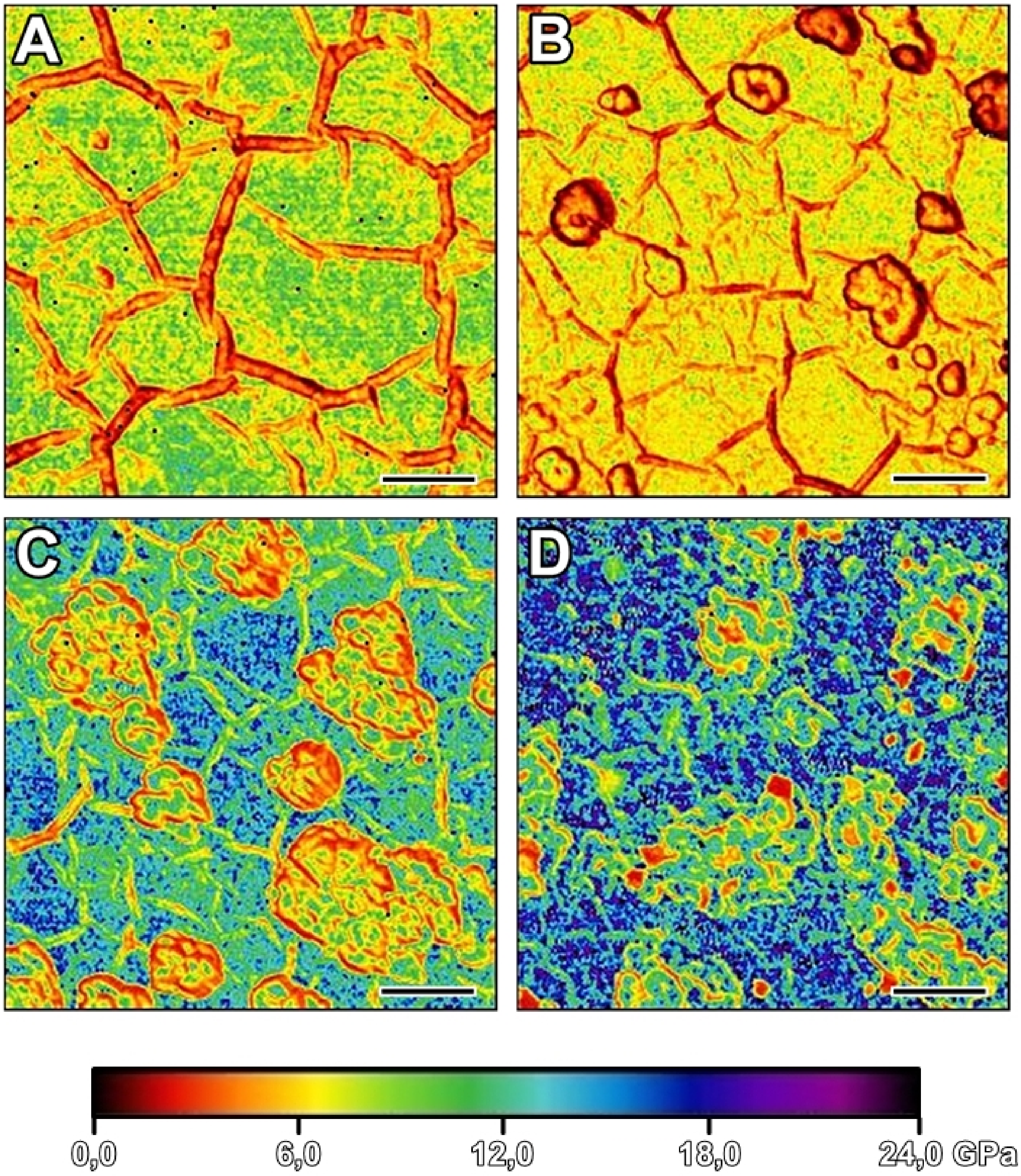

Figure S5: AFM Young's modulus images of hBAlN layers grown on sapphire substrate. a) $Al_{0.02}$; b) $Al_{0.04}$; c) $Al_{0.07}$; d) $Al_{0.13}$. The scale bars correspond to 200 nm.

Table S1: Average values of crystalite height, adhesion, deformation and stiffness modulus of hBAlN and crystaline objects obtained with atomic force microscopy analysis for studied samples.

Sample	$Al_{0.02}$	$Al_{0.04}$	$Al_{0.07}$	$Al_{0.13}$
crystalite height (nm)	-	21	27	18
hBAlN adhesion (nN)	1.49	3.89	4.31	2.98
crystalite adhesion (nN)	1.24	1.63	1.76	1.24
hBAlN deformation (nm)	0.66	1.00	0.60	0.52
crystalite deformation (nm)	1.38	1.10	0.62	0.42
hBAlN Young's modulus (GPa)	8.8	7.1	13.0	14.9
crystalite Young's modulus (GPa)	3.9	6.0	8.0	10.1

Raman spectroscopy of hBAlN samples

Raman spectra were acquired using a Renishaw inVia Raman setup equipped with a 785 nm continuous wave laser excitation source and a 100× objective. Laser power density was about 300 kW/cm^2. For each point on the sample two spectra were collected - the first with the laser beam focused on the surface of the hBAlN layer and the second with the laser beam focused inside the substrate (∼20 μm below). Then, the background spectrum was subtracted to obtain a pure epitaxial material signal.

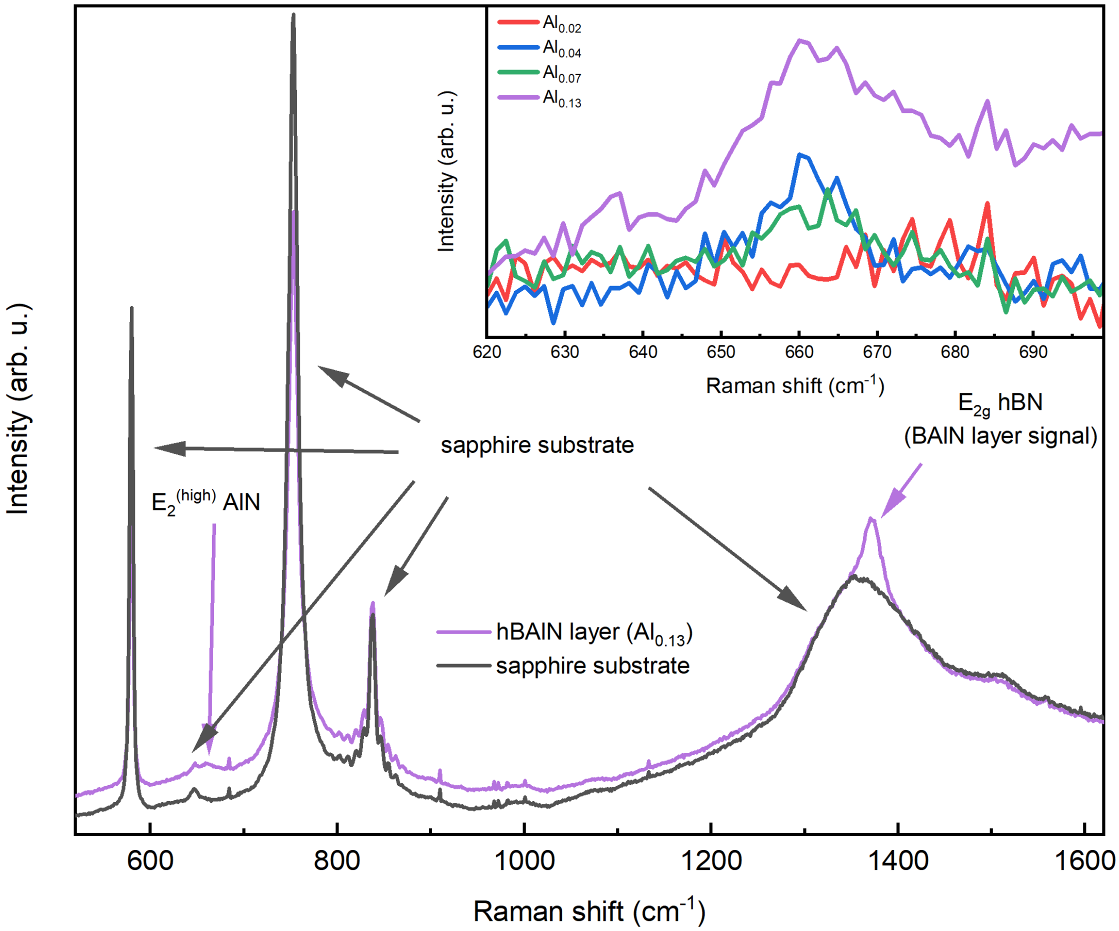

Figure S6: Exemplary Raman spectrum collected for the sample $Al_{0.13}$ with the highest TMAl:III ratio (purple line). The spectrum collected at the same point but with laser beam focused inside the sapphire substrate is presented by the gray line. The inset shows the zoom on the area related to E_2^{high} aluminum nitride mode for the samples: $Al_{0.02}$ (red line), $Al_{0.04}$ (blue line), $Al_{0.07}$ (green line) and $Al_{0.13}$ (purple line). The background was subtracted from the spectra presented in the inset.

In figure S6 we present an exemplary Raman spectrum for the $Al_{0.13}$ sample and the sapphire substrate spectrum collected at the same point. As can be seen, sapphire-related peaks overlap with the peaks for the hBAlN layer. By comparison of these two spectra we can distinguish two characteristic peaks coming from the hBAlN layer that are related to E_{2g} phonon mode in hBN ($\sim$ 1366 cm^{-1})[1] and $E_2^{(high)}$ phonon mode in AlN ($\sim$ 660 cm^{-1}).[2] The mode characteristic of AlN (sp^3-bonded material) should not be observed for samples of hBAlN (sp^2-bonded material). The presence of this signal can be explained by the crystaline objects observed in SEM (figure 4 in the main text) and AFM (figures S2-S5) images. A closer look for this spectral region is presented in the inset of figure S6. When the sapphire substrate signal is substracted from the spectra of hBAlN, the AlN-related peak can be observed more clearly. The peak intensity correlates with the amount of TMAl used in the growth process. The more TMAl the peak has higher intensity. The dependence stands in analogy to the appearance of crystaline objects observed in SEM and AFM (the more TMAl, the more crystalites) as well as detected in XRD (figure 2 in the main text). All in all implies that the objects present on the surface of hBAlN layers are composed of aluminum nitride.

To investigate the E_{2g} Raman mode of BAlN, we performed Lorentzian curve fitting. Figure S7a shows a representative spectrum for the $Al_{0.07}$ sample. As in previous analyses, the sapphire substrate signal, collected 15 μm below the surface focus inside the bulk sapphire, was subtracted. This procedure isolated the signal from the BAlN layer, which was then fitted using a Lorentzian function. From this fitting, we determined the E_{2g} Raman mode energy and the full width at half maximum (FWHM) of the peak. The mode energy values derived from Raman measurements are higher compared to those obtained from FTIR analysis, as shown in Tab. 2 of the main text, while the FWHM values are lower. These discrepancies likely stem from differences in background subtraction methods. For FTIR data, the entire spectrum is modeled directly, yielding material information without additional post-processing. Despite these differences, both techniques reveal consistent trends in FWHM for the $Al_{0.04}$, $Al_{0.07}$, and $Al_{0.13}$ samples. However, the peak position dependence

is less straightforward for the Raman data. Notably, as observed in the FTIR results, the $Al_{0.02}$ sample exhibits behavior distinct from the other samples, as discussed in the main text.

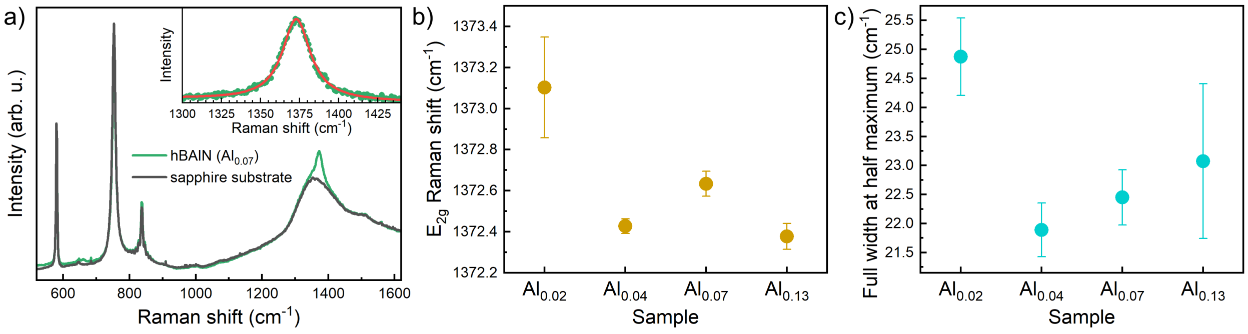

Figure S7: Analysis of E_{2g} BAlN Raman mode. a) Exemplary spectrum collected for sample $Al_{0.07}$ (green line) and sapphire substrate signal measured with the focus set 15 μm below the sapphire surface. Inset presents BAlN peak after substrate background subtraction (green dots) with fitted Lorentzian curve (red line). b) Weighted mean and standard deviation of fitted E_{2g} peak position for measured points. c) Weighted mean and standard deviation of fitted full width at half maximum (FWHM) of the peak for measured points.

HRTEM characterization of the crystalline objects

As depicted in Figure S8, the crystalline objects observed in the AFM images exhibit a crystal structure distinct from that of the hBAlN layer. Consequently, these objects are readily distinguishable in cross-sectional TEM analysis. The observed wurtzite crystal structure, confirmed by Fast Fourier Transform, is characteristic of aluminum nitride, consistent with Al_2O_3. This observation is in line with our interpretation of XRD results in the main text.

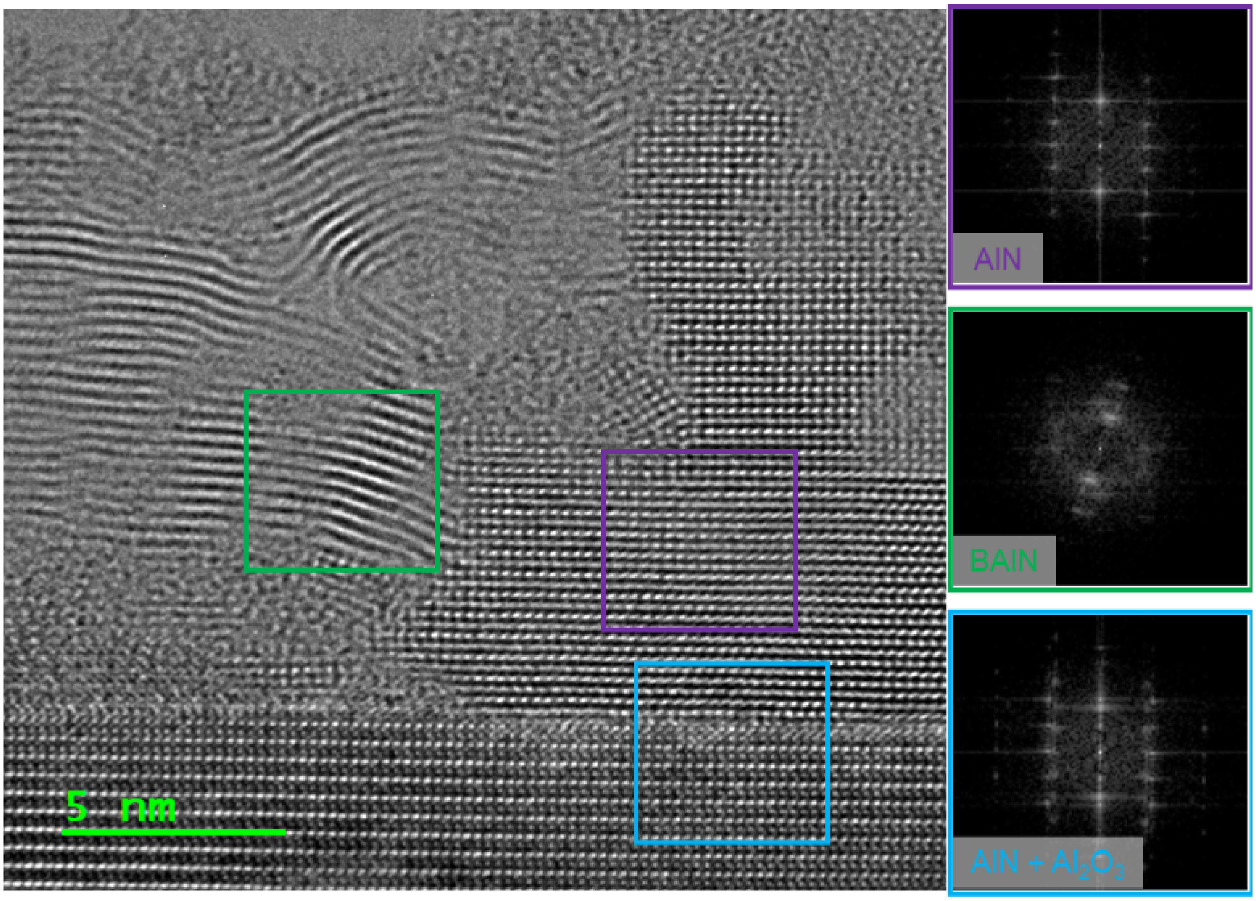

Figure S8: HRTEM result for $Al_{0.13}$ in the vicinity of crystalline object. Panels on the right are fast Fourier Transform of the area surrounded by the same color frame in HRTEM picture.

Effect of NaOH etching on absorption properties

Sample $Al_{0.13}$, featuring the largest crystalline objects, underwent etching with sodium hydroxide (pH=13) for 60 minutes. As depicted in the inset of Figure S9, AFM images post-etching revealed the removal of aluminum nitride clusters without damage to the hBAlN layer. Prior to etching, 3D structures covered approximately 27% of the sample area, whereas post-etching, the remaining darker spots covered only about 5% of the picture area. This suggests that the lateral dimensions of crystallites increase with their thickness. Importantly, after etching, the absence of AlN clusters was observed, while the two peaks in the absorption spectra remained. Furthermore, the peaks exhibited narrower widths, likely due to the reduction of inhomogeneous strains induced by the presence of ingrown wurtzite AlN. No differences were detected in the spectra near the AlN bandgap region around 6.2 eV (above the dX peak in BAlN). This can be attributed to the significantly lower absorption coefficient of wurtzite AlN, approximately 2×10^5 1/cm.[3,4] In summary, the presented results confirm that our primary observation of the two absorption peaks is attributed to the hBAlN layer itself rather than to the crystalline objects observed in AFM, SEM, and HRTEM.

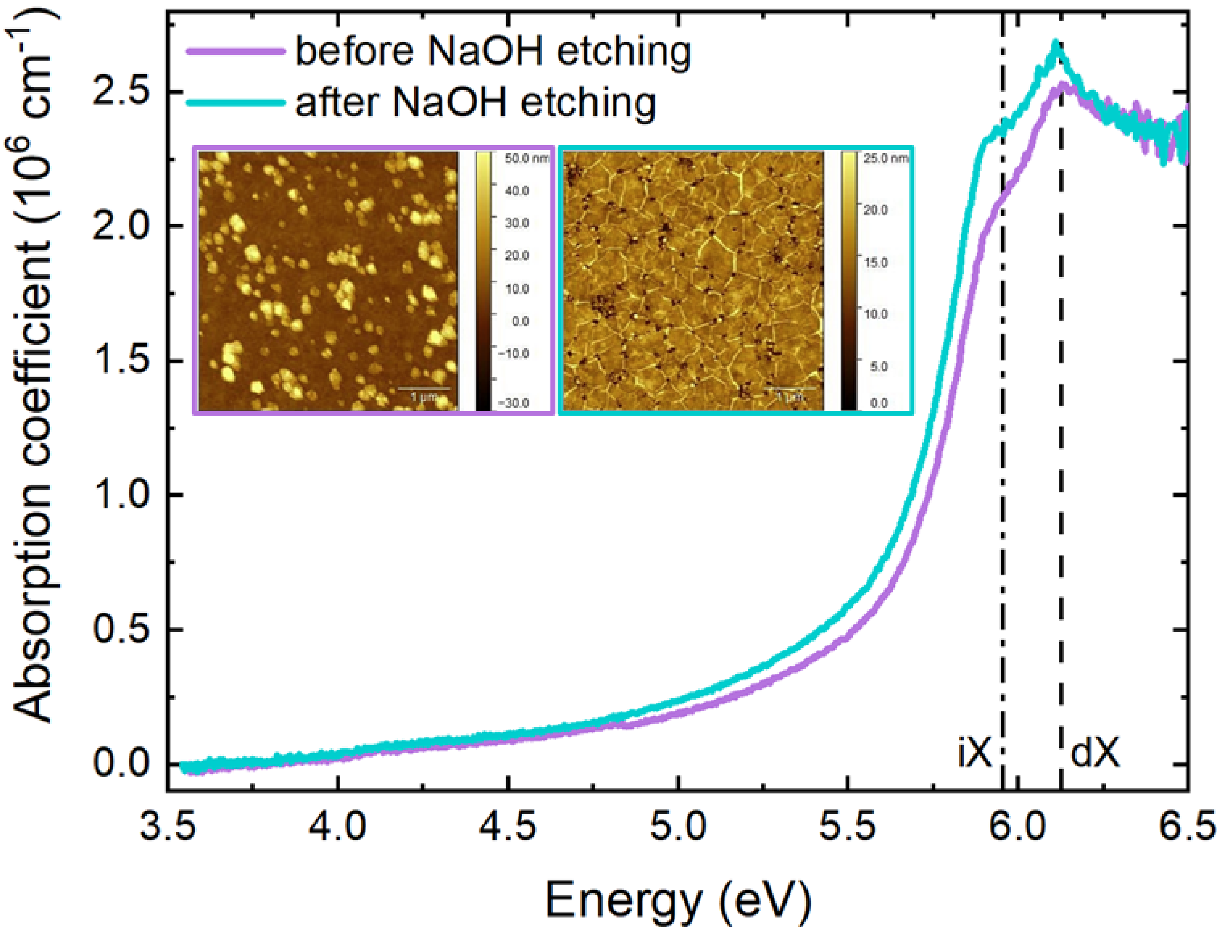

Figure S9: Absorption coefficient result for $Al_{0.13}$ before and after etching with NaOH. Black dashed and dash-dotted lines illustrate the energies of direct and indirect bandgaps in boron nitride. Inset shows AFM results of sample morphology before and after etching with NaOH.

References

(1) Kuzuba, T.; Era, K.; Ishii, T.; Sato, T. A low frequency Raman-active vibration of hexagonal boron nitride. Solid state communications **1978**, 25, 863–865.

(2) Oliveira, I.; Otani, C.; Maciel, H.; Massi, M.; Noda, L. K.; Temperini, M. L. A. Raman active E 2 modes in aluminum nitride films. Journal of materials science: materials in electronics **2001**, 12, 259–262.

(3) Yim, W. M.; Stofko, E. J.; Zanzucchi, P. J.; Pankove, J. I.; Ettenberg, M.; Gilbert, S. L. Epitaxially grown AlN and its optical band gap. Journal of Applied Physics **1973**, 44, 292–296.

(4) Perry, P. B.; Rutz, R. F. The optical absorption edge of single-crystal AlN prepared by a close-spaced vapor process. Applied Physics Letters **1978**, 33, 319–321.
